# The Case of Dr. Abner Baker, an Alleged Monomaniac, Who Was Executed on the 3d of October Last, for Killing His Brother-in-law, Daniel Bates

**Published:** 1846-01

**Authors:** 


Art. IV.—*The Case of Dr. Abner Baker, an alleged Monomaniac, who was Executed on the 3rd of October last, for killing his brother-in-law, Daniel Bates.*

We adverted briefly, in a former number, to this case. At that time it was before the Governor with a petition for pardon. The executive clemency was not exercised, and the law was suffered to take its course. The testimony given on the trial is before us, and we propose laying before our readers the material facts of the case, by which they will be enabled to make up an opinion respecting the monomania, which was insisted upon in the defence. Monomania was the only plea set up by the counsel of the prisoner; the killing was unquestionable and undenied. Baker was seen by two witnesses to ride up to the salt works where Bates was engaged, dismount from his horse, advance with a pistol, fire, and then return towards his horse. The ball was received by Bates in the back, and proved mortal in a few hours. Baker went to the house of Hugh L. White, the uncle of his wife, and stated what had occurred, appearing to be highly elated at the deed, and expressing no fears for the result if he could get justice.

The father-in-law of Baker, James White, a witness for the Commonwealth testified, that Baker, becoming jealous, had parted with his daughter three months after their marriage. Baker had charged his wife to the witness with unchastity under circumstances the most incredible; such as that she had commenced her lewd practices at nine years of age—was seduced at that early period by her teacher, a clergyman of unimpeached moral character—that Bates had come to his bed in the night and communicated with her in his presence, and that she had had criminal intercourse with several of her uncles. The witness further stated, that Baker had exhibited to him a certificate from Mrs. Baker wherein she confessed her guilt in these particulars; and that when he asked her respecting it, she said she was *obliged* to give it. Baker then proposed to witness that if he would furnish him with money he would leave the country with his daughter, go to Missouri, or some new country, and try to live with her. A sum of a thousand dollars was proposed Baker abused Bates to the witness all day; said that he was the "blackest hearted man that ever lived," and repeatedly threatened to kill him. Such language, it was testified, he used in reference to Bates on many occasions; the phrase just quoted recurs in the evidence about a dozen times; but while such was his estimate of Bates, and such his suspicions, it is important to remark that Bates, in his dying declaration, asseverated in the most solemn manner, that he was innocent of all the charges made against him, that he had never injured Dr. Baker, and that his jealousy was unfounded.

Josiah Davis, another witness for the Commonwealth, stated, that Baker, previously to his marriage, had told witness that Bates' negroes were prepared to kill him (Baker); that Bates did not treat his sister, Mrs. Bates, kindly; that he had threatened her life, and, he believed, would have killed her if his heart had not failed him; and that he stayed at Bates' house to protect his sister. Witness testified that the charges were untrue, and that Bates lived happily with his wife.

Several other witnesses on the same part swore that Baker had charged Bates to them with a conspiricy against his life, and that his negroes were bribed to murder him. It was testified that in this state of mind he left Bates' house, leaving his business behind him, as he expressed it, to avoid a difficulty; that he went to Knoxville, and after an absence of a weeks, during which time he gave strong evidences of insanity, as will presently appear, returned and perpetrated the deed over which his mind had been long brooding.

By various witnesses it was stated that Baker had manifested great apprehensions that Bates was seeking an opportunity to kill him, and had his negroes armed ready to take his life. One negro was sold, and Baker asserted to a witness that Bates disposed of him because he would not promise to engage in the murder. From all the testimony in the case it is plain, that he labored under a perpetual fear of assassination.

It was testified that Baker's eye had a peculiarly wild and fierce expression, which was increased whenever he spoke of Bates or his wife; that he was restless, and behaved in a way to alarm his friends. His step-mother related the circumstances of his behaviour at his father's, when visiting the family the first time after his marriage—his remaining nearly all the time in his room, his indisposition to see company, and his strange conversation, from all of which she came to the conclusion that he was deranged. Other members of the family bore testimony to the same point. His wife was found frequently in tears. He complained to his sisters that persons were admitted to his room at night. He suspected the chastity of his step-mother and sisters, and charged them with keeping a house of ill-fame. One individual in particular was the object of his suspicions; this man came into the room where he was passing the evening with his wife at the house of a friend, when his manner became strange, and his look wild. He paced the floor backwards and forwards, cursed and swore, and wished himself in hell. The lady of the house said to him, that he ought to be ashamed of himself; whereupon he whispered something to his wife, and she was observed to shed tears. The witness who detailed these circumstances is the mother of a deranged son, now confined in a lunatic asylum; her conviction of Dr. Baker's insanity was so clear, that she declared to his sisters and to her family that she should feel safer with her son than with Baker. All the female witnesses, as well as every man who bore testimony in the case, united in a favorable report as to the conduct of Mrs. Baker. Not a whisper was uttered against her good name, except by her unfortunate husband whose jealousy, no one doubted, was wholly groundless. From testimony now to be adduced we shall see how wild and extravagant this passion was.

After parting with his wife, and after having made an attempt upon Bates' life in a paroxysm of jealous rage, he went to Knoxville, in Tennessee, where he met two of his brothers, respectable physicians of that neighborhood. To them he related the story of all his imaginary wrongs. He said that Bates had more than once come into his room and cohabited with his wife in his presence. The particulars were minutely given, and among them he stated that Bates made a negro woman stand over him with a bowie knife while the act was in progress. He declared that there had been a plot between his wife and Bates to poison him; that on one occasion, when he sipped a little toddy made for him by his wife, without solicitation, it flew into his head and swelled it up until it felt as large as a bushel; and that if he had drunk as much as usual, "it would have blowed him to hell in five minutes." He assured his brother that his wife was pregnant when he married her, as he ascertained by a manual examination shortly afterwards, and that she miscarried while they were on their visit to his father's—a circumstance rendered utterly incredible by the testimony of all the female witnesses. He also assured him that one of their young sisters had been violated by Bates, and was then in the family way. He recited the proofs he had, that, while his wife was still indisposed from her miscarriage, she had intercourse with a man in Lancaster. To another witness he stated that the clergyman, her teacher, who seduced her, kept up his intercourse with her "until her courses came on" and then desisted—that before her marriage, while suffering from tooth ache, a man went into a room where she was lying on a bed, and cohabited with her—that a negro man, after their marriage, had crawled up to the window, got into her room, and had intercourse with her; the proof of which was, that when he slept with her "*she filled him full of seed ticks*," which had come from the negro, some of which he held out his hand to exhibit to a witness. He told the same witness that his mother had opened the door to admit a man to his bed while he was on the visit to his father's. He declared to another witness that "his wife was the smartest woman that ever lived; that she would lay her ear on his breast and ascertain when he was asleep"—and that he "wrung himself around her," and so managed to get her to give the certificates. His horse falling lame while he was at his father's, he charged a negro with having injured him for the purpose of detaining Mrs. Baker in order that he might continue to have criminal intercourse with her. After he had committed the fatal deed and was in the custody of a magistrate, he related to persons present all these transactions, and as one of them testified "told him over and over the same things with great minuteness." And when he was brought to trial before the magistrates, and the plea of insanity was set up in his defence, he declared that he "would rather be hung than that such a plea should be relied upon." He would plead justification; that he was justifiable; that Bates was at the head of a combination to take his life, and that he could prove it by Mrs. Bates.

It was made clear by all the evidence that Dr. Baker was of a remarkably suspicious disposition, and that his intimate friends had often been alarmed by evidences of insanity; still it does not appear that any of the witnesses were led to suspect the soundness of his mind by the many strange and incredible stories he told of his wife. On the contrary, being a physician in good practice, esteemed a man of strong sense, and on nearly every subject exhibiting a lucid judgment, they pretty uniformly testified their conviction that he was in his right mind. Most of them had never heard of monomania, and they had not supposed that a man might be insane on one subject while on others he evinced a sound understanding. The presiding judge, it would seem, did not admit of this distinction; for we are informed that he asked a medical witness "whether such a disease as *monomania* was known and recognized in medical authority;" and on being answered that it was both in law and medicine, he is reported to have "rejoined, in the hearing of the jury, that he had only made the inquiry as to medicine—he claimed to know something of the law himself."

Among the witnesses in this case were three physicians—Dr. H. Baker, to whose testimony we have already referred in part, Dr. Reid, and the late Professor Richardson. Concerning the state of his brother's physical system Dr. Baker stated, that "his hands were icy cold, his bowels costive, stomach irritable; that he appeared greatly distressed, watchful, and restless." But all this, and a letter received from his father intimating a fear that his son, Abner, was deranged, did not raise in his mind a suspicion that he was the subject of monomania. He showed the letter to his brother who evinced great indignation at the charge, which, we have seen, he continued to repel. He was exceedingly offended at his counsel, because they persisted in relying upon it, and never forgave his father for expressing an apprehension that he was insane. It is most worthy of remark, that, in the end, he came to regard his father and brothers as his worst enemies.

Dr. Reid testified that, in his opinion, Dr. Baker was laboring under illusions of mind in regard to his wife, and so far was deranged; that he had known B. for years, and that he was in the habit of talking wildly on some subjects; that in view of the monstrous charges he had made against his wife and others, he should certainly pronounce him deranged on that subject.

The testimony of Dr. Richardson was ample and decided. He attended the trial, by request of the friends of the accused, for the purpose of giving an opinion on the case—that opinion was, that, in all his practice for thirty years, "he had not seen a better and more interestingly developed case of monomania." So indicative was Baker's appearance of a mind diseased, that he believed he "could have picked him out of the crowd in the court house as a monomaniac." He visited the prisoner repeatedly in his jail, and found his physical condition and appearance evidently those of a deranged man; his pulse, temperature, expression of eye, hang of the muscles of his face, tongue, and whole aspect afforded to his mind infallible proofs of it. In his conversation, especially when it turned upon Bates or his wife, he was wild, incoherent, and erratic; "he seemed to believe, and asserted with a conviction apparently intuitive, things that seemed to the witness not only very incredible but impossible." When on these subjects "his eyes became singularly red, excited, and obviously maniacal."

Let us recapitulate: Dr. Baker, then, fully believed in the truth of all the charges he made, as is evident from the manner in which, time and again, and to a multitude of persons, he reiterated them. He believed that his wife was violated by a clergyman, of spotless reputation, when she was but nine years of age, that she cohabited with him until puberty, and that this clergyman made a *harem* of his school. He believed that his wife brought on abortion, a month after their marriage, by pressing on her abdomen, and that she gave birth to the child in the privy, and was able, not only to be about afterwards, but to go into company, and escape all suspicion of the females around her that she had miscarried. He believed that his mother and sisters kept a house of prostitution—that they introduced lewd men into his room at night, that, in his presence, they might violate his bed, while his wife bore the signs of her miscarriage still upon her. He believed that this wife, while yet a bride, was ready to yield, night after night, to the embraces of an aged brother-in-law, her uncles, a negro, and her own father! He not only believed that his wife was thus ready to prostitute herself to every man, of whatever color, or of whatever affinity to her, but that the intercourse took place on his own bed, and before his own eyes.

Medical men, everywhere, considering this testimony came to the conclusion expressed by Prof. Richardson, that Dr. Baker was deranged in respect to his wife, and that in a fit of insane jealousy he had taken the life of his brother-in-law. So viewing it, the physicians of Lexington, of Frankfort, of Danville, of Lancaster, and of Louisville communicated to the Governor their solemn conviction of his insanity. These expressions of professional conviction were unavailing, and the unfortunate man was executed. Under the gallows his manner was as it had been, and his vehement asseverations to the last were of his wife's infamy, and of his own justification. In other words; he died like an insane man. No relentings, no looking forward to the rewards of a hereafter, no tenderness towards the friends around him, or for his absent father and sisters, evinced the pious education he had received, or any lingerings of humanity within him.

If he had been affecting insanity up to this time, is it reasonable to believe that he would have maintained the character to the last? If not a maniac why continue to rave like one when there was no longer any hope of pardon, and while the executioner was adjusting the fatal cord about his neck? No one can doubt that the belief of his wife's baseness was rooted in his mind, and that he felt himself fully justified in taking the life of the man who had first violated his bed, and then conspired against his life. Except upon this principle, his conduct was inexplicable throughout. He had nothing to promise himself from all his cruel treatment towards his wife, and his violence towards Bates. She was the wife of his choice, and, at times, he displayed the most violent affection for her. Bates was his friend, as well as his kinsman. In his profession his prospects were fair. His father-in-law had wealth, of which, in due time, he had the prospect of becoming one of the inheritors. By kindness to his wife, and by preserving friendly relations with his wealthy and influential brother-in-law, he stood every chance to rise in his profession and do well. By taking the fatal step which he did, he brought upon himself certain ruin. It was testified by one witness, that Bates said that the doctor had quarreled with him because he did not receive money from him fast enough; but it was clear that the taking of his life would not cause it to flow faster, but, on the contrary, cut off his supply from that source altogether. The father-in-law related that Baker spoke to him of money; but it was under circumstances, and connected with accusations against a daughter of so monstrous and incredible a character, that it is impossible to read them without a feeling, that the man who would go to a father with such a story, and such a proposition must be insane. And in this light it appeared to the father, for he testified that he said to Dr. Baker, on the occasion referred to, that "he was either deranged, or one of the greatest scoundrels in the world." Up to this time Baker had stood well among men; we have no proof that he was wanting in principle, but much proof that he had been a dutiful son, and a respectable student and practitioner of medicine. By departing from a life of honor he had nothing to gain, and everything to lose. Not a shadow of evidence was adduced, that his wife was guilty of any one of his charges, and we are left to infer that the crimes imputed to her were either the hallucinations of a disordered brain, or else the fabrications of a man so base, that he was willing to render his wife infamous in the eyes of her father, and still live with her; and all this for a thousand dollars. His whole previous life is inconsistent with the latter supposition, while all that was related on the trial of his deportment after his marriage supports the idea that he was jealous even to madness. No man, not insane or utterly infamous, would have used such language concerning a wife as it is proved that this unfortunate man continued to do up to the very moment of his execution. He was not infamous; and our conclusion, therefore, is, as expressed by a multitude of medical witnesses, that he was a fit subject for a madhouse, and not the gallows.

